# The BtaF Adhesin Is Necessary for Full Virulence During Respiratory Infection by *Brucella suis* and Is a Novel Immunogen for Nasal Vaccination Against *Brucella* Infection

**DOI:** 10.3389/fimmu.2019.01775

**Published:** 2019-07-26

**Authors:** Florencia Muñoz González, Gabriela Sycz, Iván M. Alonso Paiva, Dirk Linke, Angeles Zorreguieta, Pablo C. Baldi, Mariana C. Ferrero

**Affiliations:** ^1^Cátedra de Inmunología, Facultad de Farmacia y Bioquímica, Universidad de Buenos Aires, Buenos Aires, Argentina; ^2^Instituto de Estudios de la Inmunidad Humoral (IDEHU), CONICET-Universidad de Buenos Aires, Buenos Aires, Argentina; ^3^Fundación Instituto Leloir, IIBBA-CONICET, Buenos Aires, Argentina; ^4^Centre for Ecological and Evolutionary Synthesis, University of Oslo, Oslo, Norway

**Keywords:** *Brucella suis*, bacterial adhesins, BtaF autotransporter, respiratory infection, nasal immunization, mucosal immunity, intragastric challenge

## Abstract

*Brucella* enters their hosts mostly through mucosae from where it spreads systemically. Adhesion to extracellular matrix (ECM) components or to host cells is important for the infectious process, and is mediated by several adhesins, including the BtaF trimeric autotransporter. Although Th1 responses and gamma interferon (IFN-γ) are important for protection, antibodies able to block adhesions might also contribute to prevent *Brucella* infection. We evaluated the importance of BtaF for respiratory *Brucella* infection, and characterized the immune response and protection from mucosal challenge induced by nasal vaccination with recombinant BtaF. While lung CFU numbers did not differ at day 1 p.i. between mice intratracheally inoculated with *B. suis* M1330 (wild type) and those receiving a Δ*btaF* mutant, they were reduced in the latter group at 7 and 30 days p.i. For vaccination studies the BtaF passenger domain was engineered and expressed as a soluble trimeric protein. Mice were immunized by the nasal route with BtaF or saline (control group) plus the mucosal adjuvant c-di-AMP. Specific anti-BtaF antibodies (IgG and IgA) were increased in serum, including a mixed IgG2a/IgG1 response. *In vitro*, these antibodies reduced bacterial adhesion to A549 alveolar epithelial cells. Specific IgA antibodies were also increased in several mucosae. Spleen cells from BtaF immunized mice significantly increased their IL-2, IL-5, IL-17, and IFN-γ secretion upon antigen stimulation. In cervical draining lymph nodes, antigen-experienced CD4+ T cells were maintained mainly as central memory cells. A BtaF-specific delayed-type hypersensitivity response was detected in BtaF immunized mice. Lung cells from the latter produced high levels of IFN-γ upon antigen stimulation. Although nasal immunization with BtaF did not protect mice against *B. suis* respiratory challenge, it conferred significant protection from intragastric challenge; the splenic load of *B. suis* was reduced by 3.28 log CFU in immunized mice. This study shows that nasal vaccination with BtaF+c-di-AMP protects against intragastric challenge with *B. suis* by inducing local and systemic antibody responses, central memory CD4+ T cells and strong Th1 responses. Therefore, although BtaF vaccination did not protect from *B. suis* respiratory infection, this adhesin constitutes a promising immunogen against mucosal *B. suis* infection.

## Introduction

Brucellosis is a zoonotic disease caused by bacteria of the genus *Brucella*, a Gram-negative pathogen, which affects 500,000 new people annually in the world (1, 2). In domestic animals *Brucella* causes abortion and infertility and leads to important economic losses. In contrast, human brucellosis is a debilitating disease which can evolve with chronic complications such as osteoarticular disease, meningitis, and endocarditis. Human infection can be acquired by consumption of raw meat or non-pasteurized dairy products, inhalation of infected aerosols or contact with infected animal products through conjunctiva or skin lesions (3, 4). Because of its high infectivity by the inhalatory route (5–10) *Brucella* has been included in the list of possible bioterrorism agents by the Centers for Disease Control and Prevention (CDC) (1). The entry by mucosal membranes is also important in domestic animal infection, not only by the routes already described but also by the venereal route.

*Brucella suis* is the etiological agent of swine brucellosis and one of the main human brucellosis pathogens. As the human infection is frequently acquired from animal sources, the diagnosis and vaccination of livestock are fundamental strategies to prevent human disease. However, currently there are no commercially available vaccines for use in human and swine in most countries, except in China where an attenuated strain (*B. suis* S2) is used for swine vaccination (11). Although initial reports were promising, this vaccine has not been tested in other countries. Moreover, as *B. suis* S2 is a smooth strain it induces an antibody response that precludes the serological discrimination between vaccinated and infected animals using conventional tests (12).

On the other hand, commercially available *Brucella* vaccines approved for use in cattle, goats, and sheep are based on attenuated strains, which can still produce disease in humans (13–15). In addition, they may result in abortion when administered to pregnant females, and can induce immune responses that interfere with serological diagnosis. Therefore, improved vaccines that combine safety and efficacy and can protect all susceptible animal species need to be developed (16). Acellular vaccines, such as those based on recombinant proteins, offer numerous advantages over attenuated vaccines. They are safe, well-defined, non-infectious, and cannot become virulent. However, they are frequently poorly immunogenic and require the use of adjuvants. The selection of appropriate antigens based on the knowledge about the host-pathogen interaction is essential for the success of these vaccines.

*Brucella* enters the host mostly through mucous membranes from where it spreads systemically to different organs, causing the symptoms of the disease. Adhesion of *Brucella* to extracellular matrix (ECM) components or to host cells is an important step for the infection (17–19). It was proposed that the binding of *Brucella* to host cells is mediated by molecules containing sialic acid and/or sulphated residues, and by components of the ECM such as fibronectin, collagen, and vitronectin (17, 18). Recently, we have identified several adhesins that are involved in the adhesion of *B. suis* to ECM components and host cells (19–21). One of such adhesins, the BtaF trimeric autotransporter, was involved in the adhesion to various ECM components and to human cervical (HeLa) and alveolar (A549) epithelial cells. In addition, BtaF was required for full virulence during intragastric infection in mice (20). The trimeric autotransporters are a subclass of the type V secretion systems (22, 23). They have a C-terminal domain that forms a β-barrel in the outer membrane of gram-negative bacteria, and a surface-exposed passenger (and functional) domain that predominantly forms a coiled-coil fiber (α-domain) and extends the adhesive N-terminal head from the bacterial surface (24). The adhesins, in particular those of the autotransporter families, represent attractive targets for the design of novel vaccines directed against Gram-negative bacteria (25). However, the role of the BtaF adhesin in the infection and dissemination of *B. suis* after the respiratory infection, and its potential as an immunogen in acellular mucosal vaccines against *B. suis* are currently unknown.

Since *Brucella* are intracellular facultative pathogens that establish their preferred replicative niche in macrophages (26, 27), the protective immune response against these bacteria is mainly mediated by T helper type 1 (Th1) cells that secrete gamma interferon (IFN-γ), which upregulates macrophage anti-*Brucella* activity. It has also been shown that antibodies contribute to protection against respiratory *Brucella* infection (28, 29). In particular, the induction of antibodies able to block the initial stages of infection, such as bacterial attachment to host cell components or to the ECM of the mucosal surface, might represent an attractive strategy to prevent *Brucella* infection. Therefore, while the induction of systemic immune responses following immunization represents a major goal for the ideal vaccine against brucellosis, the elicitation of a specific mucosal immune response would help to prevent pathogen entry and dissemination to other organs. To achieve this, antigens must be administered through the mucosal route with appropriate mucosal adjuvants, such as cyclic di-nucleotides.

The cyclic di-nucleotides are second-messenger molecules in bacteria that are sensed by the host *v*í*a* STING (stimulator of interferon genes) receptors, which in turn trigger several immune responses. One of these cyclic nucleotides, bis-(3′,5′)- cyclic dimeric adenosine monophosphate (c-di-AMP), has been widely shown to exert strong adjuvant activities when it is used in mucosal vaccine formulations (30–34).

Nasal vaccination usually induces appropriate antigen-specific responses not only in nasal mucosa but also in other mucosal sites, thus protecting the individual from infection through several mucosal routes. Despite the potential advantages of mucosal immunization, very few studies have evaluated the nasal route of vaccination against *Brucella* mucosal challenge. In most of these studies attenuated strains were tested, and the results were discouraging (28, 35). To our best knowledge, no studies have been published assessing the efficacy of nasal vaccination with acellular vaccines against mucosal challenge with *B. suis*. In this study, we evaluated the importance of the BtaF adhesin for respiratory *Brucella* infection, and characterized the immune response elicited by nasal vaccination with recombinant BtaF plus c-di-AMP and the protection conferred against mucosal challenge with *B. suis*.

## Materials and Methods

### Animals

Female BALB/c mice (6–8 weeks old) were purchased from Universidad Nacional de La Plata, Argentina, acclimated and randomly distributed into experimental groups. The animals were housed in a biosafety level 3 animal facility (Unidad Operativa Centro de Contención Biológica, Administración Nacional de Laboratorios e Institutos de Salud Dr. Carlos G. Malbrán, Argentina), and received water and food *ad libitum*. Experiments in mice were approved by the animal care and use committee of Facultad de Farmacia y Bioquímica, Universidad de Buenos Aires (CICUAL D N° 3300/18).

### Bacterial Strains, Culture Conditions, and Media

*Brucella suis* M1330 (wild-type strain, wt), *B. suis* Δ*btaF* and *B. suis* Δ*btaF* complemented with the *btaF* gene were grown in Tryptic Soy Broth (TSB, Bacto™) at 37°C with agitation (20). When necessary, chloramphenicol (6 μg/ml) and nalidixic acid (10 μg/ml) were added. Bacteria were washed with sterile phosphate buffered saline (PBS) and inocula were prepared on the basis of optical density (OD) readings. The actual number of colony forming units (CFU) was later determined by plating on Tryptic Soy Agar (TSA, Bacto™). All live *Brucella* manipulations were performed in biosafety level 3 facilities.

*Escherichia coli* strains used in this study, DH5α and BL21 (DE3) pLysS, were grown with agitation at 37°C in Luria-Bertani (LB) medium supplemented with kanamycin (25 μg/ml).

### Kinetics of *B. suis* Infection in a Respiratory Model

Groups of 5 female BALB/c mice were inoculated intratracheally with *B. suis* M1330 (5 × 10^4^ CFU/mice) as previously described (36) with minor modifications. Briefly, animals were anesthetized with isoflurane and after becoming recumbent, they were injected intraperitoneally with a mixture of ketamine and xylazine (100 and 10 mg/kg, respectively). Mice were placed in supine position over an acrylic backboard and restrained by the teeth using a rubber band. Under translucent illumination of the trachea, the inoculum was injected in a final volume of 20 μl in between the vocal cords with a Hamilton syringe coupled to a blunt-ended probe. Mice were euthanized at 1, 7, and 30 days post-infection (p.i.) by an intraperitoneal injection of a lethal dose of ketamine and xylazine, and their spleens, livers and lungs were aseptically removed. The whole organs were homogenized in 2 ml of sterile PBS, and serial dilutions of homogenate aliquots were plated on TSA for CFU counting.

### Virulence of *B. suis btaF* Mutant

Groups of 15 female BALB/c mice were inoculated intratracheally with 5 × 10^4^ CFU/mice of the *B. suis* strains described above (wt, Δ*btaF*, or complemented Δ*btaF*). Five mice from each group were euthanized at 1, 7, and 30 days p.i. by an intraperitoneal injection of a lethal dose of ketamine and xylazine, and their spleens, livers, and lungs were aseptically removed and homogenized in 2 ml of sterile PBS. Serial dilutions of homogenate aliquots were plated on TSA for CFU counting.

### Antigen Production

#### Molecular Cloning

All DNA manipulations were carried out using standard procedures. To construct the BtaF trimeric recombinant protein, part of the BtaF passenger domain coding sequence (BR1846) was amplified using *B. suis* M1330 genomic DNA as template, and primers BtaF_PD_F and BtaF_PD_Sint_R (5′-CTTTAAGAAGGAGATATACATATGGAGGAAAATGTTTCGCAGGTGAAACT-3′ and 5′-TCTGTTTCATGCGACCGCGGTTTTGGC-3′, respectively), thus obtaining a PCR product of 535 bp. Secondly, the GCN4tri-His sequence was amplified using plasmid pIBA-GCN4tri-His as template (37), and primers BtaF_PD_Sint_F and BatF_PD_R (5′-CCGCGGTCGCATGAAACAGATTGAAG-3′ and 5′-CGGGCTTTGTTAGCAGCCGGATCGTCGACTCAGTGATGATGATGATGATGAAGC-3′, respectively), thus obtaining a PCR product of 153 bp. Subsequently, both fragments (containing complementary regions) were ligated by overlapping PCR using the flanking oligonucleotides (BtaF_PD_F and BtaF_PD_R). The resulting fragment of 688 bp was used as a megaprimer in a PCR reaction with the pET-24a expression plasmid (Novagen) as template according to the restriction-free cloning method (38). Then, the PCR reaction product was digested with DpnI at 37°C for 2 h and the mixture was transformed into *E. coli* DH5α competent cells. Selection was carried out on LB-kanamycin plates, and the resulting plasmid (pET-BtaF-GCN4tri-His) was isolated. This construct was checked by sequencing. Finally, the pET-BtaF-GCN4tri-His plasmid was transformed into *E. coli* BL21 (DE3) pLysS competent cells.

#### BtaF Recombinant Protein Purification

Precultures of *E. coli* BL21 (DE3) pLysS bearing the pET-BtaF-GCN4tri-His plasmid were grown overnight in 5 ml of LB supplemented with 25 μg/ml kanamycin at 37°C with agitation (200 r.p.m.), and then diluted to 500 ml and grown until an OD_600_ of 0.6. At this point, protein expression was induced by the addition of isopropyl-thio-β-D-galactopyranoside (IPTG) to a final concentration of 0.5 mM, and incubation was continued for 4 h at 37°C. Cells were harvested by centrifugation at 16,000 × g for 10 min at 4°C, resuspended in binding buffer (20 mM Na phosphate buffer pH 7.4, 500 mM NaCl, 20 mM imidazole, 1 mM phenylmethylsulphonylfluoride—PMSF) and disrupted by sonication with a probe tip sonicator (QSonica-LLC, Q500), keeping bacteria on ice. Total cell lysate was centrifuged at 100,000 × g for 45 min at 4°C in a Beckman Coulter L7-65 ultracentrifuge, and the supernatant was filtrated by a 0.2 μm syringe filter. Then, it was loaded onto a HisTrap™ HP column (GE Healthcare) and elution was performed with a linear gradient of elution buffer (20 mM Na phosphate buffer pH 7.4, 500 mM NaCl, 500 mM imidazole). Purification was carried out following protein absorbance at 220 nm. A major peak was observed. The appropriate fractions were pooled and dialyzed overnight at 4°C against S200 buffer (20 mM Na phosphate buffer pH 7.4, 300 mM NaCl). The BtaF trimeric recombinant protein was further purified by Superdex 200 prep grade (GE Healthcare) in S200 buffer. The appropriate fractions were pooled and dialyzed overnight at 4°C against PBS. The quality of the final preparation was checked by SDS-PAGE (15% gel) followed by Coomassie Blue Staining. In order to separate the trimer, an aliquote of the purified protein was mixed with Laemmli sample buffer containing 3 M urea. Immunogenicity of the recombinant protein in mice was tested by Western blot. Briefly, purified BtaF recombinant protein was subjected to SDS-PAGE (15% gel) and transferred to a Hybond PVDF membrane (Amersham, GE Healthcare). Membrane was blocked with TBS 5% milk powder (w/v) with gentle agitation for 1 h at room temperature, and then probed with pooled sera from BtaF-immunized mice (see the following section), at a 1:2,000 dilution in TBS-Tween 0.05 and 1% milk, with gentle agitation at 4°C for 16 h. Membrane was then incubated with goat HRP-conjugated anti-mouse (1:30,000) secondary antibody (Santa Cruz) in TBS-Tween 0.05 and 1% milk, with gentle agitation at room temperature for 2 h. The blot was developed using ECL Prime (Amersham, GE Healthcare) following the manufacturer's instructions, and was imaged using an ImageQuant LAS4000 Molecular Imager (GE Healthcare). Protein folding was confirmed by Circular Dichroism (CD Spectrometer Jasco J-815). The theoretical molecular weight of the trimer, 66.6 kDa, was predicted using the ProtParam tool from ExPASy (39). The molecular weight of the native protein in solution was confirmed by Static Light Scattering (Precision Detectors PD2010 90° light scattering instrument) tandemly connected to a high-performance liquid chromatography, a LKB 2142 differential refractometer, and to a 486 Absorbance Detector (Waters) set at 220 nm. The purified protein (500 μl, ~0.5 mg/ml) was loaded into a Superdex 75 GL 10/300 (GE Healthcare) column, and the chromatographic run was performed in Phosphate Buffer Saline pH 7.4 and 250 mM sodium chloride under isocratic conditions at a flow rate of 0.4 ml/min at 20°C (room temperature). The molecular weight was calculated by relating its 90° and RI signals and comparison of this value with the one obtained for bovine serum albumin (BSA, molecular mass: 66.5 kDa) as a standard using the software Discovery32.The purified protein was incubated with polymyxin B-Sepharose (Thermo Fisher Scientific, Massachusetts, USA) overnight at 4°C with agitation to eliminate lipopolysaccharide (LPS) contamination. The protein concentration of the antigen preparations was determined by the bicinchoninic acid method (Pierce, Rockford, IL) using BSA as standard. All BtaF preparations used contained <0.1 endotoxin units per mg of protein.

### Nasal Immunization

To evaluate the effect of nasal vaccination with the BtaF protein, 3′5′-c-di-AMP (c-di-AMP) (InvivoGen, California, USA) was used as a mucosal adjuvant. Mice were divided into two groups (*n* = 5) and were immunized once a week for 3 weeks by nasal instillation with BtaF (10 μg) plus c-di-AMP (10 μg), or saline plus c-di-AMP (10 μg) in a final volume of 20 μl. Immunization times were selected on the basis of previous studies (40, 41). At 0 and 21 days, after the first immunization, serum samples were obtained to evaluate levels of specific anti-BtaF antibodies. One week after last immunization, saliva, feces, bronchoalveolar lavage fluid (BAL), vaginal lavage fluid, lung homogenates, and spleens were obtained for immunological studies.

### Determination of Antibody Response

BtaF specific antibodies were measured by indirect ELISA. Specific IgG, IgG1, IgG2a, and IgA antibodies were determined in serum samples obtained at 0 and 21 days after the first immunization, and specific IgA was measured in saliva, feces, BAL, vaginal lavage fluid, and lung homogenates. In all cases, polystyrene plates (Corning Incorporated, New York, USA) were coated with purified BtaF recombinant protein (0.5 μg/well) in PBS during 1 h at 37°C. After this incubation, plates were washed three times with PBS containing 0.05% Tween-20 (PBS-T) and blocked overnight at 4°C with 200 μl of PBS containing 3% of skim milk. Plates were incubated with appropriate dilutions of the different samples for 2 h at room temperature and then were washed three times with PBS-T. Isotype-specific goat anti-mouse horseradish peroxidase conjugates (Sigma Aldrich, Missouri, USA; Jackson ImmunoResearch, Pennsylvania, USA) were added at appropriate dilutions. After 1 h of incubation at 37°C, plates were washed three times and TMB substrate solution (BD TMB Substrate Reagent Set, BD Bioscience, San Diego, USA) was added to each well. After 15 min of incubation at room temperature, the reaction was stopped by the addition of 2N H_2_SO_4_, and the OD was measured at 450 nm in a microplate reader (Multiskan). Cut-off values for the ELISA assays were calculated as the mean specific OD plus 3 SD from sera from non-immunized mice. Serum titers were established as the reciprocal of the last dilution with an OD higher than the cut-off.

### Inhibition Assays

The capacity of antibodies to inhibit bacterial adhesion to epithelial cells was evaluated. *B. suis* M1330 was grown overnight in TSB at 37°C with agitation and washed with sterile PBS. The adequate volume of bacterial suspension, providing a multiplicity of infection (MOI) of 100 for the subsequent cellular infection, was incubated with decomplemented sera from immunized or control mice (both at 1/10 dilution) for 1 h at 37°C with gentle shaking. After incubation, the bacterial suspension was used to infect a confluent monolayer of A549 human lung epithelial cells (ATCC CCL185) at a MOI of 100 in 96-well plates (5 × 10^4^ cells/well). The cell culture was washed with sterile PBS and total bacteria associated with the cells were determined by lysis with 0.2% Triton X-100 after 1 h of incubation at 37°C in 5% CO_2_ and plating serial dilutions. To quantify the number of intracellular viable bacteria, the infected monolayers were incubated in the presence of 100 μg/ml gentamicin (Sigma Aldrich) to kill extracellular bacteria (42). The number of adherent bacteria was calculated as the difference between total bacteria associated to the cells and intracellular bacteria.

The ability of antibodies to inhibit bacterial adhesion to previously described BtaF ligands was also assayed. The assay was performed essentially as described previously for testing bacterial adhesion (20), except that bacteria were incubated with anti-BtaF sera before ligand interaction. Briefly, 96-well plates (Nunc Maxisorp) were coated overnight at 4°C with 50 μl of 100 μg/ml solutions of each ligand (hialuronic acid, type I collagen or fetuin) dissolved in PBS, and were then washed three times with PBS to eliminate unbound ligand. Bacteria were grown overnight, washed, and resuspended with the serum dilutions to achieve a final concentration of 1 × 10^9^ CFU/ml. Pooled sera from each immunization group (BtaF+c-di-AMP or c-di-AMP, 21 days post-immunization) and from non-immunized mice (pre-immunization samples) were used (1:10 dilution in sterile PBS). After 1 h incubation at 37°C, 50 μl of bacterial suspensions were added to each ligand-coated well and incubated at 37°C for 3 h. After incubation, wells were washed three times with PBS to remove non-adherent bacteria, and were then incubated with 0.05% trypsin−0.5% EDTA for 10 min at 37°C to harvest adherent bacteria. Serial dilutions of the bacterial suspensions were done and plated on TSB agar for CFU counting.

### Opsonophagocytosis Assay

*Brucella suis* was grown overnight in TSB, washed with sterile PBS and resuspended in a 1:10 dilution of pooled sera from each immunization group (BtaF+c-di-AMP or saline+c-di-AMP) or from non-immunized mice. After 1 h incubation at 37°C, the bacterial suspension was used to infect murine macrophages (RAW 264.7 cell line) at a MOI of 100 in 96-well plates (5 × 10^4^ cells/well) for 2 h at 37°C (time 0 p.i.). Macrophages were used either untreated or pretreated for 24 h with recombinant murine IFN-γ (100 IU/ml). The cell culture was washed with sterile PBS and cultured with complete medium containing 50 μg/ml of gentamicin for 2 h to kill extracellular bacteria. To determine the number of intracellular bacteria cells were washed three times with sterile PBS and lysed with 0.2% Triton X-100, and serial dilutions of the lysates were plated on TSA for CFU counting.

### *In vitro* Cellular Responses

One week after last immunization, mice were euthanized with a lethal dose of ketamine and xylazine (400 and 32 mg/kg, respectively) and spleens and lungs were removed. Lung cells were obtained after incubation with 200 U/ml type IV collagenase supplemented with 5% Fetal Bovine Serum (FBS) and 20 U/ml Deoxyribonuclease I for 20 min at 37°C. Lung and spleen cells were cultured in a 96 wells plate at 1 × 10^6^ cells/well or 48 wells plate in duplicate at 4 × 10^6^ cells/ml, respectively, in RPMI 1640 supplemented with 10% FBS, 1 mM pyruvate, 2 mM L-glutamine, 100 U/ml penicillin and 100 μg/ml streptomycin. Lung cells were stimulated with 10 μg/ml BtaF protein or complete medium alone, while spleen cells were stimulated with 10 μg/ml BtaF protein, 5 μg/ml Concanavalin A (ConA), or complete medium alone. After 72 h of incubation at 37°C and 5% CO_2_, cell culture supernatants were collected and gamma interferon (IFN-γ), interleukin 2 (IL-2), IL-5, and IL-17 production was analyzed by commercial sandwich ELISA, according to the manufacturer's instructions (BD Bioscience).

### Flow Cytometry Analysis

Cervical lymph nodes cells (1 × 10^6^ cells/well) from immunized and control mice were cultured in 96-well plates with BtaF (10 μg/ml) in RPMI 1640 supplemented with 10% FBS, 1 mM pyruvate, 2 mM L-glutamine, 100 U/ml penicillin, and 100 μg/ml streptomycin for 16 h at 37°C and 5% CO_2_. After stimulation, cells were stained with APC-anti-mouse CD3 monoclonal antibody (mAb) (clone 17A2, Thermo Fisher Scientific), PE-Cy™5-anti-mouse CD4 mAb (clone RM4-5, BD Bioscience), PE-Cy™7-anti-mouse CD8 mAb (clone 53-6.7, BD Bioscience), PE-anti-mouse CD44 mAb (clone IM7, BD Bioscience), and FITC-anti-mouse CD62L mAb (clone MEL-14k, BD Bioscience). Flow cytometry analysis was performed using FACSAriaII flow cytometer (BD Bioscience) and further analyzed using FlowJo 7.5 software (TreeStar Inc.).

### Delayed-Type Hypersensitivity (DTH) Test

One week after last immunization, mice were injected intradermally in one footpad with 10 μg of BtaF in 10 μl of PBS, and in the contralateral footpad with an equal volume of PBS as negative control. The footpad thickness was measured 48 and 72 h later by using a digital caliper. At each time point, the mean increase in footpad thickness was expressed as the relation between the BtaF footpad and the saline footpad.

### Protection Assessment

At 15 days after last immunization, mice immunized with BtaF plus c-di-AMP and the control group (saline) were challenged through the intratracheal or the intragastric route with 6.5 × 10^4^ or 4 × 10^7^ CFU of *B. suis* M1330, respectively. Three weeks after challenge, mice were euthanized, and lungs and spleens (for the intratracheal challenge) or the spleens (for the intragastric challenge) were aseptically removed. Dilutions of homogenized organs were plated on TSA and incubated for 3 days at 37°C. The number of CFU was counted and results were represented as the mean CFU/ml ± SEM per group.

### Statistical Analysis

Data were analyzed using analysis of variances (ANOVA). Multiple comparisons between all pairs of groups were made with the Tukey's post-test, and those against a control group were made with Dunnett's post-test. A *p* < 0.05 was considered as statistically significant. All statistical analyses were performed with the GraphPad software (San Diego, CA).

## Results

### Kinetics of *B. suis* Infection in Lungs and Dissemination to Peripheral Organs

**Figure 1:**
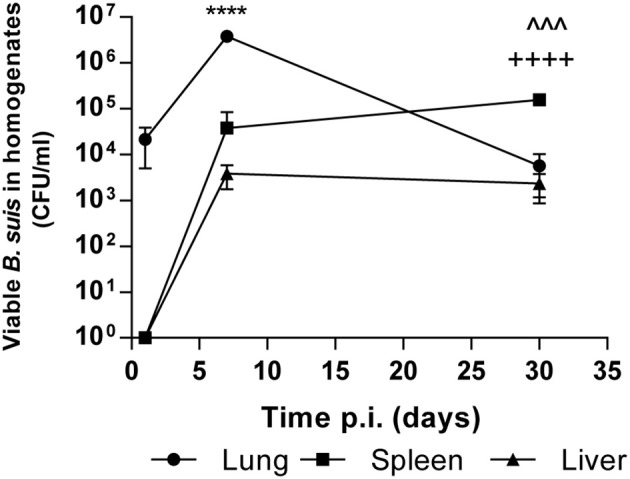
Kinetics of intratracheal infection with *B. suis*. BALB/c mice were infected with 5 × 10^4^ CFU/mice of *B. suis* wt. At 1, 7 and 30 days post-infection mice were euthanized, and the lungs, spleens, and livers were removed. Dilutions of organs homogenates were plated and CFU were counted. Values are means ± SD of duplicate measurements from three independent experiments. Asterisks indicate significant differences between 1 and 7 days and up arrowheads and crosses significant differences between 7 and 30 days. ****p < 0.0001; ^∧∧∧^*p* < 0.001; ^++++^*p* < 0.0001.

In order to evaluate the impact of BtaF on the course of respiratory *B. suis* infection, we first characterized the mouse model of intratracheal infection with *B. suis* at early (1 and 7 days) and later times (30 days) post-infection (p.i.). At these time points CFU numbers were determined in lung, liver, and spleen homogenates. As shown in Figure 1, the pulmonary bacterial burden increased significantly (1.55 log) during the first week p.i. but significantly decreased at 30 days p.i. (1.81 log as compared to 7 days p.i.). *B. suis* could disseminate from the initial infection site and was first recovered from spleen and liver at 7 days p.i. The bacterial load in spleen significantly increased at 30 days p.i. as compared to previous days (*p* < 0.001, *T*-test vs. 7 days p.i.). However, the bacterial burden in liver remained constant (*p* > 0.05).

### BtaF Is Required for Full Virulence of *B. suis* in an Intratracheal Infection

It was previously shown that BtaF is required for a successful infection of *B. suis* administered through the oral route in mice (20). To evaluate if BtaF has a similar impact in a respiratory model of infection, groups of 15 mice were anesthetized and inoculated through the intratracheal route with *B. suis* wt, the Δ*btaF* mutant, or the Δ*btaF* complemented strain. Five mice from each group were sacrificed at 1, 7 and 30 days p.i., which represent early and stabilized infection times, and the bacterial burden in spleen, lungs and liver was measured. At 1 day p.i., no differences in lungs burden were found between the wt strain and the Δ*btaF* mutant (data not shown).

**Figure 2:**
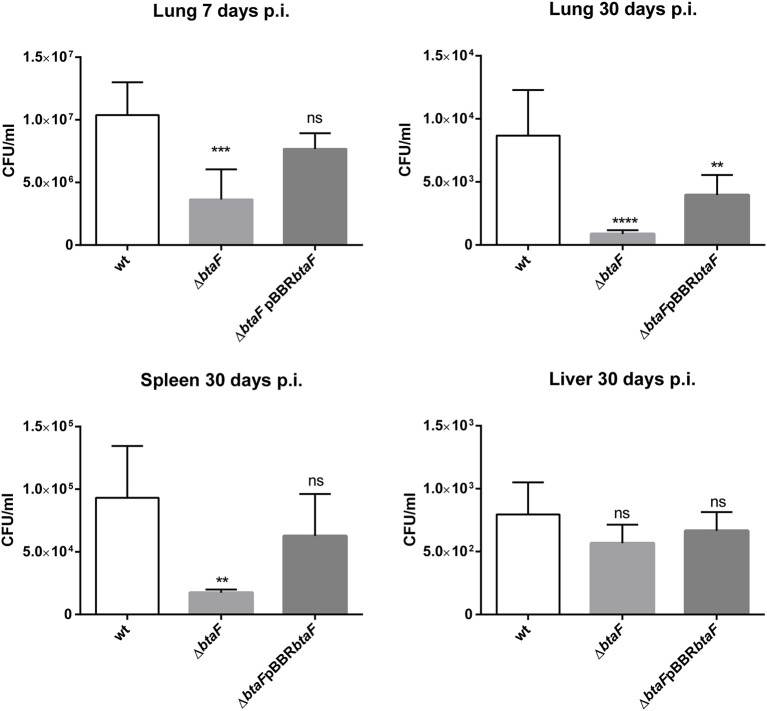
Role of BtaF in *B. suis* intratracheal infection. BALB/c mice were inoculated by the intratracheal route with *B. suis* wt, *B. suis* Δ*btaF*, or the complemented strain. Mice from each group were euthanized at 7 and 30 days post-infection, and the lungs, spleens, and livers were removed. Dilutions of organs homogenates were plated and CFU were counted. Values are means ± SD of duplicate measurements from three independent experiments. **p < 0.01, ***p < 0.001, and ****p < 0.0001 vs. wild type strain n.s., non-significant.

At 7 and 30 days p.i., the lung infection with *B. suis* Δ*btaF* was reduced by 0.28 log and 1 log, respectively, compared with the wt strain (Figure 2). Although no differences in CFU counts were detected in spleen at 7 days (data not shown), a reduction of 0.54 log was observed in this organ at 30 days p.i. with *B. suis* Δ*btaF*. No differences were observed in liver colonization between the wt and mutant strains. In all cases, no statistical differences were observed between the wt and complemented strains, except for lung tissue at 30 days p.i., in which only partial complementation was observed.

These results show that BtaF is involved in *Brucella* lung colonization and dissemination to spleen and is required for full virulence of *B. suis* in mice infected through the intratracheal route.

### Nasal Immunization With BtaF Plus c-di-AMP Elicits Systemic and Mucosal Humoral Immune Responses

**Figure 3:**
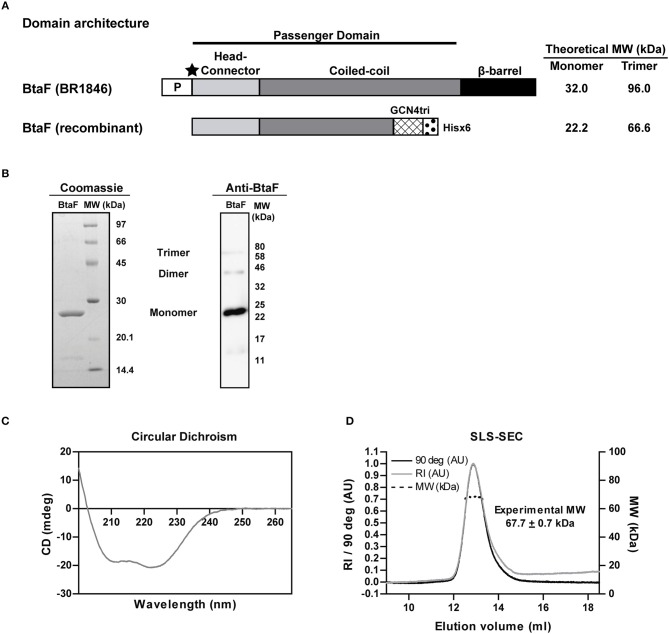
Properties of the *B. suis* BtaF recombinant protein. Schematic representation of the amino acid sequence of BtaF wt of *B. suis* (BR1846) and BtaF recombinant protein **(A)**. P: signal peptide; the black star indicates the predicted cleavage site of the signal peptide, MW, Molecular Weight. Purified BtaF recombinant protein was subjected to SDS-PAGE and subsequent Coomassie Blue Staining (left), and also to Western blot using pooled sera from BtaF-immunized mice (right) **(B)**. Circular Dichroism spectrum of BtaF recombinant protein **(C)**. Oligomeric state of BtaF recombinant protein in solution. BtaF was subjected to SEC coupled to a light-scattering instrument connected in tandem to a differential refractometer detector. BtaF molecular weight was estimated by the relation of scattering/RI **(D)**.

**Figure 4:**
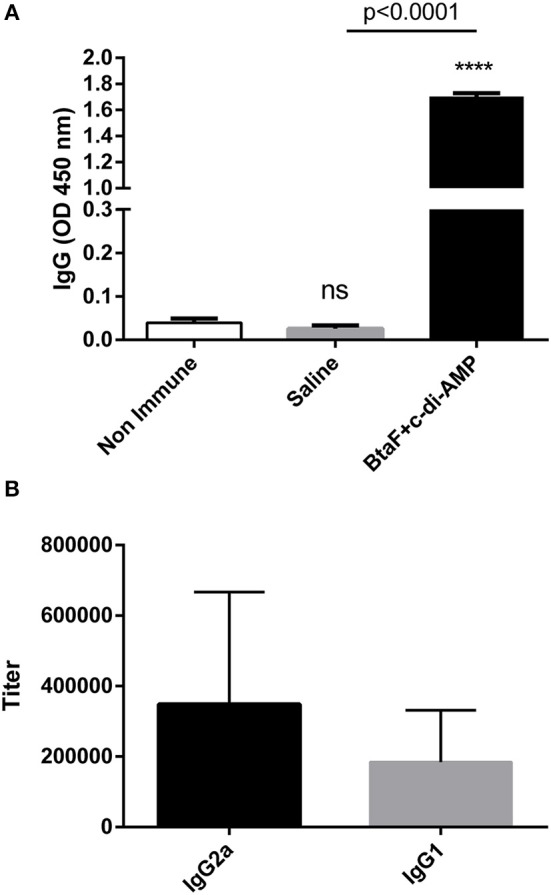
Serum levels of BtaF specific antibodies after intranasal immunization. Balb/c mice were immunized with BtaF+c-di-AMP or saline+c-di-AMP at days 0, 7, and 14. Blood samples were collected at day 21 to determine anti-BtaF IgG, IgG1, and IgG2a antibody levels by ELISA. Results are expressed as the mean OD_450nm_ + SD **(A)** or as titers **(B)** of values determined in duplicate for three independent experiments. Cut-off values for the ELISA assay were calculated as the mean specific OD_450nm_ + 3SD obtained for sera from non-immunized mice. Serum titers were established as the reciprocal of the last dilution yielding an OD_450nm_ higher than the cut-off. ****p < 0.0001, and n.s. non-significant *vs*. non-immune.

**Figure 5:**
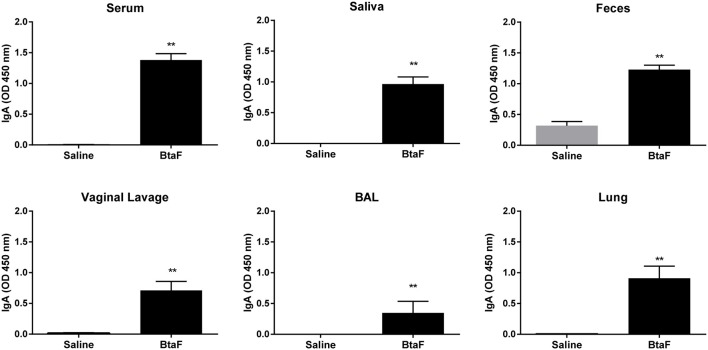
Anti-BtaF IgA antibodies in sera and mucosal samples. BALB/c mice were immunized with BtaF+c-di-AMP (BtaF) or saline+c-di-AMP (saline) at days 0, 7, and 14. One week after last immunization serum, saliva, feces, vaginal lavage, and bronchoalveolar (BAL) fluids and lung homogenates were obtained, and specific IgA antibody levels were measured by ELISA. Results are expressed as the mean OD_450nm_ + SD of three independent experiments. The asterisks indicate significant differences between the immunized group and the saline group. **p < 0.01.

Given the role of BtaF in *B. suis* infection through the respiratory route, we decided to evaluate the BtaF potential as an antigen for nasal vaccination. To achieve this aim, we designed, expressed and purified most of the BtaF passenger domain as a soluble trimeric protein. The GCN4tri sequence was fused to the C-terminal portion of BtaF in order to help the protein to form a trimer (37) and keep its native conformation (Figure 3A). Analysis through SDS-PAGE of the two step purified BtaF trimeric recombinant protein showed that the protein used in the following immunizations was highly purified (Figure 3B, left panel). Moreover, Western blot analysis indicated that the protein was immunogenic in mice (Figure 3B, right panel). Circular dichroism indicated that the secondary structure of BtaF is mainly α-helix (Figure 3C), and Static Light Scattering coupled to a Size-Exclusion Chromatography (SEC-SLS) indicated that recombinant BtaF had an experimental molecular weight of 67.7 ± 0.7 kDa in solution, which is similar to the theoretical molecular weight for the trimer of 66.6 kDa estimated by the ProtParam tool from ExPASy (39) (Figure 3D). To evaluate if BtaF is a potential antigen for nasal vaccination, mice were separated into two groups (*n* = 5) and were immunized by the nasal route with BtaF (10 μg) plus c-di-AMP (10 μg) or saline plus c-di-AMP (10 μg) at days 0, 7, and 14. Specific anti-BtaF antibodies were measured in different samples. Significant differences in anti-BtaF IgG (*p* < 0.0001 *vs*. non-immune; *p* < 0.0001 *vs*. saline) (Figure 4A) and anti-BtaF IgA levels (*p* < 0.01 *vs*. saline) (Figure 5), were detected at 21 days after first immunization, reaching median titers of 204,800 and 12,800, respectively. To further investigate the humoral response induced by nasal administration of BtaF, the titers of specific serum IgG subclasses (IgG1 and IgG2a) were determined. As shown in Figure 4B, a mixed Th1-associated IgG2a and Th2-associated IgG1 response was observed in the sera of BtaF immunized mice.

On the other hand, nasal immunization with BtaF+c-di-AMP triggered a significant induction of specific mucosal IgA antibody production in saliva, feces, vaginal lavage, BAL, and lungs compared with the control group (saline+c-di-AMP) (Figure 5).

**Figure 6:**
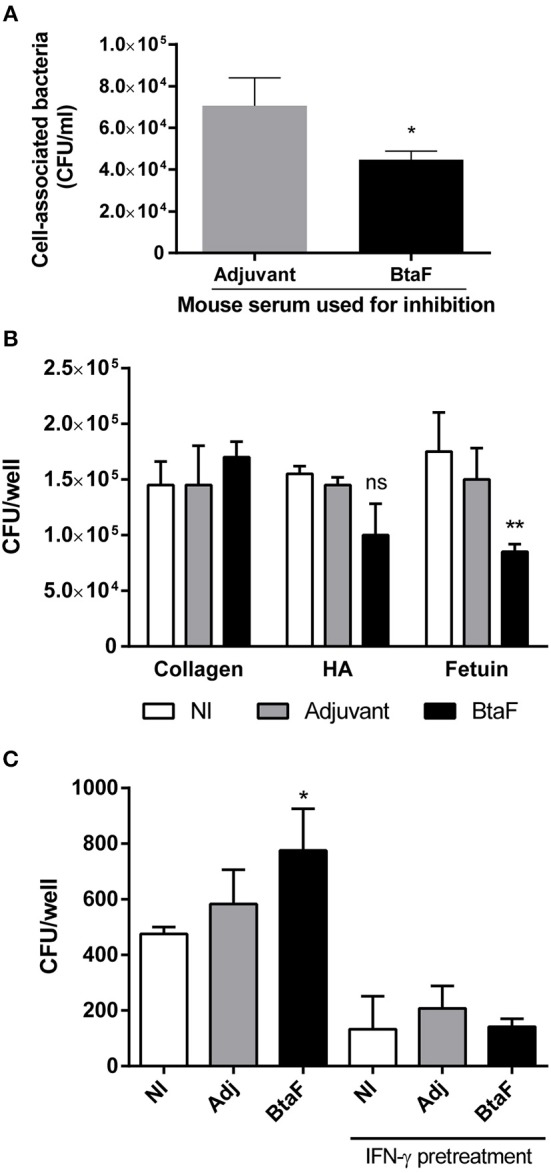
Neutralizing and opsonizing capacity of BtaF-specific antibodies. *B. suis* wt was incubated with serum from mice immunized with either BtaF+c-di-AMP (BtaF) or saline+c-di-AMP (Adjuvant) before co-culture with a confluent monolayer of A549 cell line. Total bacteria associated to the cell monolayer were determined **(A)**. Similarly, bacteria were incubated with sera from mice of the BtaF+c-di-AMP (BtaF) or saline+c-di-AMP (Adjuvant) immunization groups or from non-immunized mice (NI) before addition to wells coated with type I collagen, hialuronic acid (HA) or fetuin. Adherent bacteria recovered after trypsinization were determined **(B)**. Bacteria were preincubated as described and added to murine macrophages for 2 h. Macrophages were used either untreated or pretreated for 24 h with recombinant murine IFN-γ. After treatment with gentamicin for 2 h to kill extracellular bacteria, cells were lysed and the lysates were plated for CFU counting **(C)**. Results are expressed as mean ± SD of duplicate measurements from three independent experiments. *p < 0.05, **p < 0.01 vs. Adjuvant group **(A)** or NI group **(B,C)**.

To analyse the functionality of the serum specific antibodies elicited by nasal immunization, its capacity to inhibit bacterial adhesion to epithelial cells and to previously described BtaF ligands was evaluated. Serum antibodies from BtaF+c-di-AMP immunized mice could significantly reduce bacterial adhesion to A549 epithelial cells in comparison with serum from the control group (Figure 6A). In addition, these antibodies were also able to reduce significantly the binding of *B. suis* to fetuin and also reduced, albeit non-significantly, its binding to hialuronic acid (Figure 6B). *In vitro*, the phagocytosis of *B. suis* by murine macrophages was significantly increased when bacteria were preincubated with sera from the BtaF+c-di-AMP group as compared to preincubation with sera from non-immunized mice (Figure 6C). In contrast, preincubation with sera from the saline+c-di-AMP group had no effect. IFN-γ-activated macrophages killed internalized bacteria at similar levels regardless of the preincubation serum.

### Nasal Immunization With BtaF Plus c-di-AMP Induces BtaF-Specific Cellular Immune Responses

**Figure 7:**
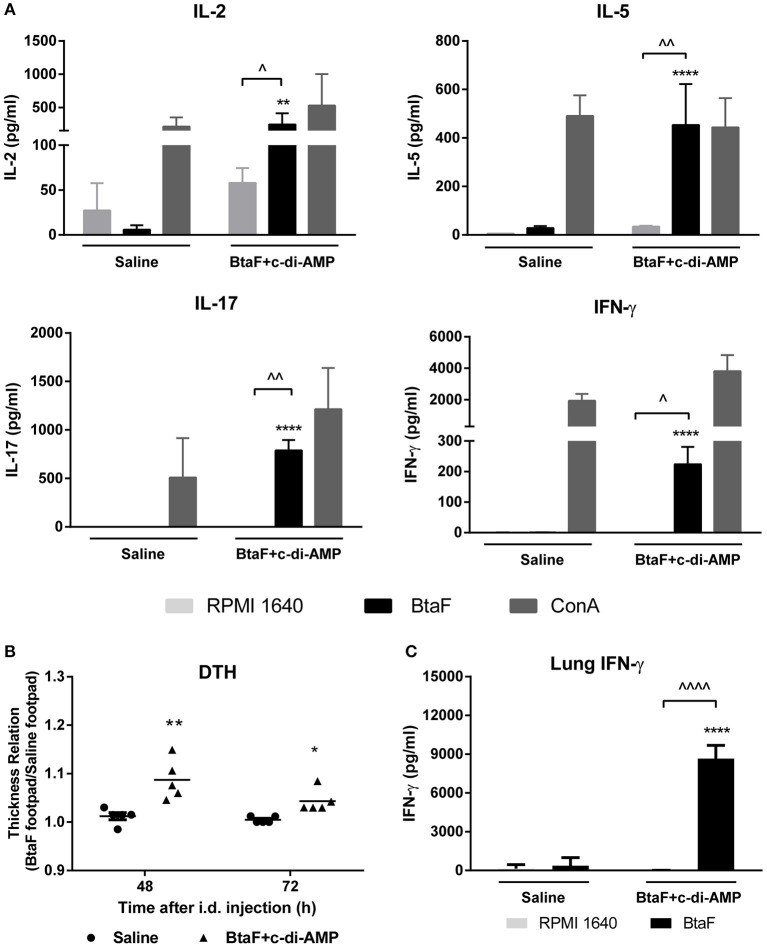
Specific cellular immune response in BtaF immunized mice. Lung cells and splenocytes from immunized and control mice were cultured in duplicate with BtaF, ConA, or medium. At 72 h post-stimulation culture supernatants were harvested from splenocytes to measure levels of IL-2, IL-5, IL-17, and IFN-γ **(A)**, and from lung cells to measure IFN-γ **(C)** by ELISA. Results are expressed as the mean concentration (pg/ml) ± SD of duplicate measurements from three independent experiments. Up arrowheads indicate significant differences between BtaF and control condition (medium) in the vaccinated group (^∧^*p* < 0.05; ^∧∧^*p* < 0.01; ^∧∧∧∧^*p* < 0.0001). Asterisks indicate significant differences between vaccinated and control mice for the same stimulus (**p < 0.01 and ****p < 0.0001). To perform the DTH test **(B)**, 1 week after last immunization mice were injected intradermally in one footpad with 10 μg BtaF and in the contralateral footpad with an equal volume of saline. The footpad thickness was measured 48 and 72 h later. The mean increase in footpad thickness was expressed as the relation between the BtaF footpad and the saline footpad. *p < 0.05 and **p < 0.01 *vs*. saline at 48 and 72 h, respectively.

It has been demonstrated that the cellular immune response is important in protection against *Brucella* infection (43–45). Thus, in order to characterize the cellular immune response elicited by nasal immunization with BtaF, lung and spleen cell suspensions from BtaF immunized and control mice were cultured *ex vivo* with BtaF or with complete culture medium alone (RPMI) as control. As shown in Figure 7A, spleen cells from BtaF immunized mice stimulated *ex vivo* with BtaF secreted significantly higher levels of IL-2, IL-5, IL-17, and IFN-γ compared with the control group. ConA, used as a positive control, induced the production of the corresponding cytokines in all groups.

To corroborate the induction of BtaF-specific Th1 responses, as suggested by the results described above, the delayed type hypersensitivity (DTH) response was determined in immunized mice. One week after last immunization, mice were injected intradermally in one footpad with BtaF and in the contralateral footpad with an equal volume of saline as negative control. As shown in Figure 7B, a BtaF specific DTH response was observed at 48 and 72 h in BtaF immunized mice. Interestingly, the stimulation of the lung cells with BtaF induced a high production of IFN-γ when compared with the cells of the control group (Figure 7C). This result suggests that the nasal vaccine induces a strong cellular specific immune response in lung against the BtaF antigen. Altogether these results indicate that nasal immunization with BtaF plus c-di-AMP induces specific cellular immune response, both at the mucosal and systemic level.

### Administration of BtaF Plus c-di-AMP Promotes Ag-Experienced Effector CD4+ T Cells

**Figure 8:**
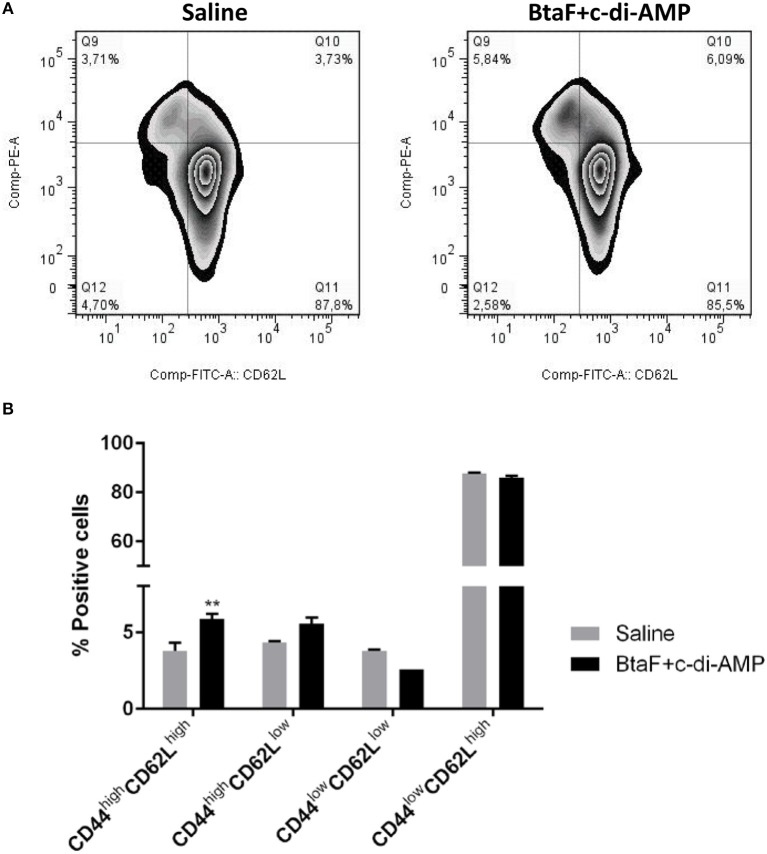
Phenotypic analysis of memory CD4+ T cells by flow cytometry. Cervical lymph nodes cells (1 × 10^6^ cells/well) from immunized and control mice were cultured in 96-well plates with BtaF (10 μg/ml) in complete medium for 16 h. The percentage of CD4+ T cells expressing the CD62L and CD44 markers was determined by flow cytometry. Subpopulations determined for each experimental group are shown in representative dotplots **(A)**, and their mean proportions (±SD) are shown in the bar graph **(B)**. **p < 0.01 vs. saline.

To determine whether T cells from immunized mice develop into memory T cells or maintain an activated phenotype, the Ag-experienced T cells were identified by the expression of CD44 (46, 47) in cervical draining lymph nodes from immunized mice after *in vitro* re-stimulation. Memory T cells can be further divided based on CD62L (L-selectin) expression into central memory T cells (CD62L^high^) and effector memory T cells (CD62L^low^) (48). Then, the percentage of cells expressing CD44 and the activation marker CD62L was analyzed in the CD4+ T cell population (Figure 8A).

Phenotypic analysis showed that CD4+ T cells from mice nasally immunized with BtaF+c-di-AMP exhibited a significantly higher proportion of the CD44^high^/CD62L^high^(5.86%) phenotype and a tendency, albeit not significant, toward a higher percentage of CD44^high^/CD62L^low^ (5.54%) subpopulation as compared to cells from control group (3.77 and 4.32%, respectively; Figure 8B). These results indicate that Ag-experienced CD4+ T cells are maintained mainly as central memory cells in mice immunized with BtaF plus c-di-AMP.

### Nasal Immunization With BtaF Plus c-di-AMP Confers Protection Against Intragastric Infection

Nasal immunization with BtaF+c-di-AMP elicited Ag-specific cellular and humoral immune responses both systemically and at mucosal surfaces. To assess if such immune responses conferred protection against respiratory challenge, immunized mice were infected through the intratracheal route with virulent *B. suis* wt, and 3 weeks post-challenge the lung and splenic loads of bacteria were determined. As shown in Table 1, immunization with BtaF protein did not confer any protection against *B. suis* respiratory challenge, as no significant differences were observed in organs colonization between immunized and control groups.

**Table 1:** Protection from respiratory and intragastric challenge.

	**Saline (log CFU/ml, mean ± SD)**	**BtaF (log CFU/ml, mean ± SD)**	**Protection level (log)**	**Significance**
**Intratracheal challenge**				
Lung	3.841 ± 0.168	3.843 ± 0.213	0.002	n.s.
Spleen	4.653 ± 0.114	4.538 ± 0.194	0.115	n.s.
**Intragastric challenge**				
Spleen	4.471 ± 0.622	1.185 ± 0.386	3.285	*P* < 0.0001

*Mice from BtaF+c-di-AMP and saline groups were challenged through the intratracheal or intragastric route with B. suis wt. CFU were measured in lungs and/or spleens at 20 days post-challenge. Results are expressed as mean ± SD of duplicate measurements from two independent experiments*.

Taking into account that we previously demonstrated that BtaF is required for full virulence after intragastric infection (20) and that in the present study nasal immunization with BtaF generated a humoral immune response in the gastro-intestinal mucosae, we evaluated the potential protection conferred by nasal immunization with BtaF against intragastric challenge with *B. suis* wt. As shown in Table 1, at 3 weeks post-challenge immunized mice showed a significant clearance (*p* < 0.0001) of *B. suis* from spleen compared with the control group, achieving a 3.28 log reduction in CFU counts. These results indicate that nasal immunization with BtaF recombinant protein induces protection against *B. suis* infections acquired through the gastro-intestinal mucosae.

## Discussion

Nasal vaccination is a desirable method to induce immunity against infectious diseases in humans as well as animals. Not only it is easily administered compared to other immunization methods, as it does not need needles, but it also induces mucosal as well as systemic immunity. Although *Brucella* infection is acquired through mucosal routes, brucellosis is a systemic disease. After adhesion and penetration of the epithelial barrier, bacteria spread to the reticuloendothelial system, and affect different organ systems. Therefore, an effective nasal brucellosis vaccine must induce mucosal immunity able to stop *Brucella* at the mucosal surface, and must also induce systemic immunity to eliminate the infected cells.

Nasal vaccines based on live attenuated *Brucella* strains have been tested in some studies to evaluate protection against mucosal infection, and were ineffective (28, 35). In addition, the risk of human infection and illness caused by attenuated strains prevents its use in humans. Although swine brucellosis is a worldwide distributed zoonotic disease, there are no vaccines approved for use in humans or pigs to protect against infection caused by *B. suis*. Moreover, there are no approved vaccines to prevent human infections by any *Brucella* species. In this study, we evaluated the role of the BtaF adhesin from *B. suis* during intratracheal infection in the mouse model and evaluated the protection capacity of BtaF recombinant protein as a novel acellular vaccine against mucosal challenge. Designing strategies that interfere with bacterial adhesion to host components may also help to prevent disease progression.

The inhalation of contaminated aerosols with *Brucella* spp. is an important cause of human and animal brucellosis. Most studies about respiratory infection have focused on infections by *B. abortus* and *B. melitensis*, with only one study describing a mouse model of infection with aerosolized *B. suis* (49). Despite the valuable information provided by that study, there is no knowledge regarding bacteria replication and systemic dissemination during the first week after *B. suis* airborne infection. In this study we used a murine model of intratracheal infection with *B. suis* to investigate the importance of the BtaF adhesin at early and late time points post-infection. After intratracheal infection, the pulmonary load of *B. suis* wt increased 1.55 log during the first week, but decreased to almost the initial inoculum at 30 days p.i. The pathogen could disseminate early from the initial infection site in the lungs, since it was recovered from spleen and liver at 7 days p.i. We recently demonstrated that *B. abortus* persists in the mouse lung without modifying its initial load during the first week after intratracheal infection (36). These results differ from those obtained in the present study and demonstrate that *B. suis* causes a more acute infection than *B. abortus* in the lung. When mice were infected with the *B. suis* Δ*btaF* mutant no differences in lung burden were observed as compared with the wt strain at 1 day p.i. (data not shown). However, at later times there was a significant decrease in the burden of bacteria in lungs and spleen in animals infected with the mutant strain as compared to those infected with the wt strain. These results agree with those of a previous study showing that the deletion of BtaF reduces the splenic load of *B. suis* in mice infected by the intragastric route (20). Recently it was shown that after being inhaled, most *Brucella* bacteria are captured by the alveolar macrophages (50). In the present study, BtaF deletion did not affect the adherence and infection of *B. suis* to primary murine alveolar macrophages (data not shown). Our results suggest that after being inhaled, most of the *B. suis* bacteria are phagocytosed by alveolar macrophages, and that BtaF does not have a relevant role in this interaction. In line with these results, it was previously shown that the adhesion and intracellular survival of *B. suis* in murine macrophages was not affected by the absence of the BmaC adhesin (19). At later times after intratracheal infection, the BtaF adhesin could participate in the interaction of the bacterium with other lung non-phagocytic cells, such as alveolar epithelial cells. We have previously demonstrated that *B. suis* adheres and infects human alveolar epithelial cells (A549 cell line) (51) and that BtaF deletion reduces bacterial adhesion to these cells (20). Moreover, BtaF participated in the resistance of *B. suis* to the bactericidal activity of complement, an important component of an efficient immune response against certain pathogens. This resistance could explain the impairment of Δ*btaF* mutant to achieve the same burden as the wt strain in peripheral organs and the incapability to efficiently persist in lungs. Together our results show that the BtaF adhesin is required for full virulence of *B. suis* after intratracheal infection.

Taking into account the relevance of the BtaF adhesin in the persistence and dissemination of *B. suis* after inoculation by mucosae, in this study we evaluated the immunogenic and protective potential of the recombinant BtaF adhesin as a nasal subunit vaccine. BtaF is a trimeric autotransporter protein that contributes to adhesion to epithelial cells and many components of the ECM (20). The BtaF recombinant protein was designed to be expressed as a soluble trimeric protein without the C-terminal β-barrel translocator domain. Using the BtaF domain that is exposed on the bacterial cell surface in its native (and trimeric) structure, combined with the appropriate adjuvant (see below), was probably an important factor to achieve a protective immune response against *B. suis*. In line with this, some proteins from the autotransporter families were shown to be promising antigens for the design of vaccines against other pathogens, including *Bordetella pertussis* (52, 53), *Haemophilus influenza* (54), *Haemophilus ducreyi* (55), *Shigella* (56), and Enterotoxigenic *Escherichia coli* (ETEC) (57). In all these cases, a recombinant protein corresponding to the autotransporter passenger domain has been used, reinforcing the potential of this protein family as subunit vaccines.

Subunit vaccines require a good immunogen and an appropriate adjuvant. In this study, mice were vaccinated by the nasal route with BtaF recombinant protein plus c-di-AMP. Several studies demonstrated that c-di-AMP, a cyclic di-nucleotide, exerts strong adjuvant activities when it is used in mucosal vaccine formulations. c-di AMP is able to activate dendritic cells, promoting both local and systemic immune responses and stimulating a balanced T helper (Th1/Th2/Th17) immune response (30–32, 34). Moreover, administration of c-di-AMP by mucosal route also induces a strong humoral immune response accompanied by secretory IgA detected both locally, as well as at distant mucosal sites (32, 33). In line with these previous reports, high levels of anti-BtaF IgG and IgA were detected in serum of BtaF plus c-di AMP immunized mice, which could also partially reduce *B. suis* adhesion to A549 cells. The fact that only a partial reduction was achieved probably relates to the existence of numerous adhesins on the surface of *B*. *suis* (19–21), which may confer redundant binding capacities to the pathogen. In particular, BmaC and BtaE adhesins have been shown to mediate *B. suis* adhesion to A549 cells. In addition, anti-BtaF sera were also able to reduce significantly the bacterial binding to fetuin, a previously described BtaF ligand (20). Of note, the phagocytosis of *B. suis* by murine macrophages was enhanced by the previous opsonization of the bacterium with serum antibodies against BtaF. Since IgG subclass responses are determined by the pattern of cytokines secreted by CD4+T cells, we measured the titers of antigen-specific IgG1 and IgG2a antibodies. BtaF-vaccinated mice exhibited a mixed IgG1/IgG2a response to BtaF. IgG2a antibodies are important for defense as they activate in phagocytes a broad spectrum of antimicrobial responses (e.g., opsonization and release of inflammatory mediators). In addition, nasal immunization elicited a significant production of specific IgA antibodies at the respiratory, gastrointestinal and genital mucosae. These antibodies could contribute to reduce the initial adhesion of the bacteria to the mucosal epithelium.

Due to its intracellular residence, the protective immune response against *Brucella* requires cell-mediated-immunity, which includes IFN-γ-producing CD4+ T cells (58), while the role played by CD8+ cells in protection is less clear. Some studies concluded that CD8+ T cells are critical for the resolution of infection, whereas others suggested that they are dispensable (44, 59, 60). In our study, nasal vaccination with BtaF plus c-di-AMP induced a Th1 response that could be measured *in vivo* and *in vitro*. Splenocytes and lung cells of immunized mice produced high levels of IFN-γ after *in vitro* stimulation with the antigen, indicating the generation of a Th1 response both locally and systemically. To further characterize the T cells involved in the cellular responses to BtaF, the antigen-experienced T cells were identified by the expression of the memory marker CD44 and the lymph node homing molecule CD62L. BtaF vaccinated mice exhibited a low but significant increase in the antigen specific central memory CD4+ T cell population in cervical lymph nodes. Moreover, nasal vaccination with BtaF plus c-di-AMP also induced the Th17 subset. The strong production of IL-17 by the spleen cells of BtaF vaccinated mice may be associated to both the adjuvant (32–34) and the mucosal route of administration (50, 60–62).

To determine whether the immune response triggered by our vaccine can protect against *Brucella* infection, vaccinated and unvaccinated mice were challenged through the intragastric route with *B. suis* M1330. The criterion used to determine if a vaccine induces a protective response against *Brucella* spp. is the reduction of the bacterial load in spleen of vaccinated mice as compared to unvaccinated mice (61, 63, 64). The BtaF vaccine proved to exert high levels of protection against intragastric *B. suis* infection. This vaccine reduced splenic colonization by 3.28 log in challenged mice, which is a high level of protection as compared to other experimental *Brucella* vaccines based on single antigens. Although the protective mechanisms against *B. suis* oral infection are not well-established, previous studies showed that Th17 cells have a protective role in oral RB51 (vaccine strain) and also in recombinant unlipidated Omp19 mice vaccination against *B. abortus* oral infection, suggesting that Th17 cells may act synergistically with Th1 cells to achieve protection through vaccination (61, 62). The protection conferred by the BtaF nasal vaccine could be due to both the humoral immune responses elicited in the gastrointestinal mucosa as well as the mixed Th1/Th17 profile generated during the immunization.

Unlike what was observed for oral infection, nasal vaccination with BtaF did not protect against *B. suis* respiratory infection. No differences were observed in bacterial load in spleen or lung between vaccinated and unvaccinated mice after intratracheal challenge. Recent studies suggest that the immune response necessary to protect against *Brucella* spp. depends on the type of vaccine and its composition, the route of vaccination and the route of challenge (59–62). In the case of pulmonary brucellosis, protection is determined mainly by the delivery method and vaccine composition (28, 35, 60). Parenteral vaccination against brucellosis proved ineffective for brucellae clearance from lungs (65, 66). Several studies assayed the efficacy of nasal vaccination with attenuated strains currently used for livestock parenteral vaccination. Nasal vaccination with RB51 did not induce respiratory protection against nasal *B. abortus* challenge (35). Similarly, nasal administration of *B. abortus* S19 vaccine did not induce significant clearance of *B. abortus* from spleen upon intranasal challenge but induced significant clearance of bacteria from lungs (35). The most successful approaches regarding protection against pulmonary infection consisted in oral and nasal administration of high doses (10^9^ CFU) of inactivated or attenuated strains (60, 63) that implies, in the case of attenuated strains, the risks associated with its manipulation, stability and administration.

Apparently, according to our results, the activation of specific humoral and cellular immune responses in the site of entry and the generation of memory T cells are sufficient to protect against oral infection but not against respiratory infection with *B. suis*. Currently little is known about the immune response needed to protect the lungs from a respiratory infection by *Brucella*. Recently, Clapp et al. (60) demonstrated that nasal *B. melitensis* Δ*znuA* vaccination protects the lung from *B. melitensis* respiratory infection, and that CD8+ T cells, but not CD4+ and Th17 cells, are essential for this protection. In contrast with this study, Yinst et al. (59) demonstrated that CD8 knockout mice are protected from nasal challenge after oral vaccination with a live attenuated strain of *B. melitensis*.

In conclusion, our results demonstrate that nasal vaccination with BtaF plus c-di-AMP confers protection against intragastric challenge with *B. suis* by inducing a specific humoral immune response both locally and systemically, central memory CD4+ T cells, and a mixed T helper response with a strong induction of the Th1 phenotype. This is the first demonstration of a nasal subunit vaccine capable to elicit a high level of protection in mice infected through the oral route. In addition, according to the results of this study, it is unlikely that a single antigen could confer protection against respiratory infection with *Brucella* spp. An approach to identify antigens that induce synergistically protective immune responses must be considered.

## Data Availability

The datasets generated for this study are available on request to the corresponding author.

## Ethics Statement

Experiments in mice were approved by the animal care and use committee of Facultad de Farmacia y Bioquímica, Universidad de Buenos Aires (CICUAL D N° 3300/18).

## Author Contributions

FM, MF, GS, DL, PB, and AZ conceived and designed the experiments. FM, GS, MF, and IA performed the experiments. FM, MF, GS, DL, AZ, and PB analyzed the data. FM, MF, GS, AZ, and PB wrote the manuscript.

### Conflict of Interest Statement

The authors declare that the research was conducted in the absence of any commercial or financial relationships that could be construed as a potential conflict of interest.
